# Polymyxin Delivery Systems: Recent Advances and Challenges

**DOI:** 10.3390/ph13050083

**Published:** 2020-04-29

**Authors:** Natallia V. Dubashynskaya, Yury A. Skorik

**Affiliations:** Institute of Macromolecular Compounds of the Russian Academy of Sciences, Bolshoy pr. V.O. 31, St. Petersburg 199004, Russia; dubashinskaya@gmail.com

**Keywords:** polymyxin, colistin, drug delivery, drug carriers, antimicrobial resistance, bioavailability

## Abstract

Polymyxins are vital antibiotics for the treatment of multiresistant Gram-negative ESKAPE pathogen infections. However, their clinical value is limited by their high nephrotoxicity and neurotoxicity, as well as their poor permeability and absorption in the gastrointestinal tract. This review focuses on various polymyxin delivery systems that improve polymyxin bioavailability and reduce drug toxicity through targeted and controlled release. Currently, the most suitable systems for improving oral, inhalation, and parenteral polymyxin delivery are polymer particles, liposomes, and conjugates, while gels, polymer fibers, and membranes are attractive materials for topical administration of polymyxin for the treatment of infected wounds and burns. In general, the application of these systems protects polymyxin molecules from the negative effects of both physiological and pathological factors while achieving higher concentrations at the target site and reducing dosage and toxicity. Improving the properties of polymyxin will be of great interest to researchers who are focused on developing antimicrobial drugs that show increased efficacy and safety.

## 1. Introduction

The World Health Organization published an R&D list pertaining to antibiotic-resistant bacteria and the measures required to address the global increase in resistance to antimicrobial drugs, including resistance to carbapenems and third-generation cephalosporins. The most critical group of microbes are the multidrug-resistant Gram-negative bacteria, such as *Acinetobacter*, *Pseudomonas,* and various *Enterobacteriaceae* (including *Klebsiella*, *Escherichia coli*, and *Serratia*), as these can cause severe lethal infections (bacteremia and pneumonia) [1].

The need is great for innovation in antibiotic research; however, the development of new antimicrobial agents is complicated due to the regulatory environment and financial risks [2]. One alternative strategy to the synthesis of new drugs is the development of nanomedicine-based forms of existing antimicrobial drugs to control multidrug resistance of microorganisms [3,4,5,6,7,8,9,10,11]. One group of drugs that has seen a revival in medical practice, given the multidrug resistance of Gram-negative bacteria (the so-called ESKAPE pathogens *Enterococcus faecium*, *Staphylococcus aureus*, *Klebsiella pneumoniae*, *Acinetobacter baumannii*, *Pseudomonas aeruginosa,* and *Enterobacter*) and the absence of new antibiotics, are the old antimicrobial drugs, the polymyxins. Polymyxins have significant therapeutic potential because they are effective against four of the six ESKAPE pathogens (*K. pneumoniae*, *A. baumannii*, *P. aeruginosa,* and *Enterobacter)* [12,13,14,15,16,17,18,19,20,21,22,23,24].

At present, several reviews have addressed delivery systems for polypeptide antibiotics [25,26], and still, others have presented the pharmacological characteristics of polymyxins and the clinical experience of their use for the treatment of multidrug-resistant infections [13,27]. This review focuses on recently developed polymyxin delivery systems and analysis of their effectiveness for different routes of administration.

## 2. Chemical Structure of Polymyxins

Polymyxins are a group of polypeptide antibiotics that includes several chemically different compounds (polymyxin A–E, M, etc.). Of these, only polymyxin B and polymyxin E have clinical applications [13,28,29].

**Figure 1:**
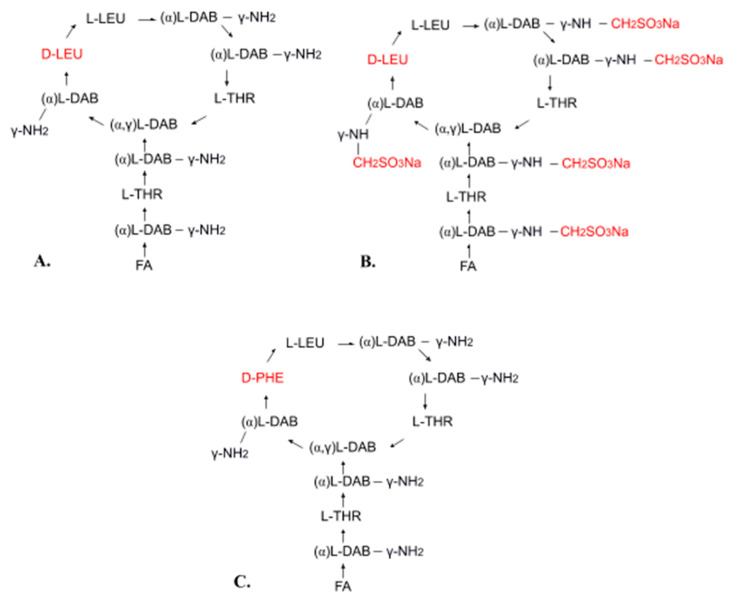
Chemical structure of polymyxin E (**A**), sodium colistimethate (**B**), and polymyxin B (**C**): FA—fatty acid, THR—threonine, LEU—leucine, DAB—α,γ-diaminobutyric acid, PHE—phenylalanine, α and γ indicate the amino groups forming the peptide linkage.

Polymyxin E (colistin) is a mixture of related decapeptides, polymyxin E1 (colistin A) and polymyxin E2 (colistin B). These have a general structure composed of a cyclic heptapeptide moiety and a side chain acylated at the N-terminus by a fatty acid (Figure 1A). Polymyxins E1 and E2 contain the same amino acids but differ in their fatty acid components (6-methyl-octanoic acid and 6-methyl-heptanoic acid, respectively). Different pharmaceutical formulations can contain different amounts of these two components [13,30,31,32,33,34,35]. For pharmaceutical use, polymyxin E is available in the form of colistin sulfate (topical and oral use) and as sodium colistimethate (injection and inhalation). Sodium colistimethate is a derivative of colistin methanesulfonic acid and is characterized by the presence of methanesulfonic acid in the sodium salt form on each amino group of the five diaminobutyric acid moieties (Figure 1B) [36].

Polymyxin B is also used in clinical practice. It differs from polymyxin E in its phenylalanine content (Figure 1C). Both polymyxin E and polymyxin B have the same mechanism of action and similar applications, but polymyxin B has less activity and causes more common and severe toxicity [13,37]. Polymyxin B is used for injection in the form of polymyxin B sulfate [13,37].

## 3. Mechanism of Action and Side Effects

Polymyxins exert their bacteriostatic effect by damaging the membrane of the bacterial cell. Polymyxins are selective for Gram-negative bacteria, since the primary molecular target of these antibacterial agents is the three-domain lipopolysaccharide (lipid A, oligosaccharide, and O-antigen) that is the main component of the outer membrane of Gram-negative bacteria [38,39]. This lipopolysaccharide has several negative charges with which polymyxins can bind by electrostatic interactions. This binding competitively replaces membrane-stabilizing ions, such as Ca^2+^ and Mg^2+^, thereby destabilizing the lipopolysaccharide layer and allowing the introduction of the polymyxin hydrophobic chain into the hydrophobic domain (lipid A). As a result, the permeability of the outer membrane increases and the polymyxin penetrates into the periplasmic space (through the process of self-promoted uptake) [40,41]. In the periplasmic space, polymyxin forms contacts between the outer and internal membranes and promotes the fast and selective exchange of anionic phospholipids. This, in turn, causes an osmotic imbalance and ultimately the death of the bacteria. Thus, lipid A is the main target for polymyxins, and the hydrophobic interactions between polymyxin and the membrane lipids are important factors in the mechanism of action of this antibiotic. In fact, one of the mechanisms of bacterial polymyxin resistance is related to lipid A modifications or to the complete loss of lipopolysaccharides, as these changes prevent this key interaction of polymyxins with the outer membrane of Gram-negative bacteria [42,43,44,45].

Importantly, the polymyxin docking with the bacterial membrane is not related to any specific proteins (receptors) but is instead a simple electrostatic interaction of a cationic molecule with a negatively charged membrane. For this reason, bacterial resistance to polymyxins is relatively rare compared to resistance to other antibiotics. Therefore, polymyxins are currently the last-line antibiotic for multidrug-resistant Gram-negative infections [46,47,48,49,50,51,52]. Polymyxin resistance is mainly caused by structural remodeling of lipid A, located on the bacterial cell surface, via biochemical and genetic mechanisms. This remodeling is initiated by the actions of membrane-associated enzymes (ArnT, EptA, and AlmEFG). The phenotypic resistance to polymyxins is divided into two types: chromosomally encoded intrinsic resistance and transposon/plasmid-mediated transferable resistance related to MCR-like enzymes (MCR-1). Different plasmids can mediate the rapid transfer of MCR-1 among various species of bacteria, and this becomes a key factor in the development of global polymyxin resistance [53,54,55,56].

In addition, various pathogens, such as polymyxin-sensitive *Salmonella* species, are able to penetrate into macrophages, where they persist and multiply within the cell compartments. Several nanomaterials, including nanospheres, liposomes, and mesoporous silica nanoparticles with surfaces modified by specific cell ligands, are able to penetrate into macrophages and kill these pathogens [57]. Metal-based nanoparticles, including gold nanoparticles, are particularly promising platforms for the intracellular delivery of antimicrobial peptides [58].

One strategy for overcoming microbial resistance to polymyxins is the use of adjuvants, such as anthelmintic salicylanilides like niclosamide, oxyclozanide, rafoxanide, and closantel [59,60,61,62]. Another is the use of polymyxin-based combination therapy with carbapenems, rifampicin, β-lactams, vancomycin, fosfomycin, and tigecycline [42,63,64,65,66,67].

Polymyxin is of critical medical importance because it is effective against selected Gram-negative bacteria (including *Acinetobacter* species, *P. aeruginosa*, *Klebsiella* species, and *Enterobacter* species, *E. coli, Salmonella* species, *Shigella* species) that cause severe nosocomial multidrug-resistant infections, including nosocomial pneumonia. Polymyxins are formulated for parenteral use (for treatment of cystic fibrosis, pneumonia, bacteremia, and urinary tract infections), inhalation (cystic fibrosis, pneumonia), and topical use (otic and ophthalmic solutions). Colistin sulfate and colistimethate sodium are not absorbed after oral administration, so they can only be used for the treatment of gastrointestinal tract (GIT) infections [13,68,69,70,71,72,73,74,75]. The most common polymyxin side effects are nephrotoxicity and neurotoxicity, and both renal and neurological toxicity are considered to be dose-dependent [13,76,77,78,79].

In summary, polymyxins are clinically important antibiotics, but their use is limited to severe site reactions because of their toxicity. The development of targeted and prolonged-release delivery systems that would reduce the dose and frequency of administration, and thereby reduce the polymyxin toxicity, is, therefore, a critical medical need. In addition, polymyxins are essentially not absorbed by the GIT, so their encapsulation into suitable carriers would greatly improve intestinal permeability, thereby imparting a new oral bioavailability for these important drugs.

## 4. Polymyxin Delivery Systems

The use of suitable nanomedicine-based devices can improve drug delivery and overcome the disadvantages of various routes of administration. Utilization of polymeric particles, or liposomes for intravenous administration, allows control of the residence time of the drug in the systemic circulation due to sustained release, as well as ensuring targeted delivery. The polymeric particles, conjugates, and liposomes in the form of inhalation formulations protect drugs from the aggressive environment of the inflamed lung (high salt concentrations, changes in oxygen levels), improve interaction with mucus, help overcome mucociliary clearance, and contribute to the penetration of antibiotics into the bacterial biofilm. At the same time, a higher concentration of the drug in the lungs is achieved than can be obtained with intravenous administration, leading to a faster pharmacological effect. In addition, solid polymeric particles and liposomes have suitable sizes (1–5 μm, and less than 1 μm) for antibiotics to reach the lower airways and infected foci by inhalation administration. Consequently, these drug carriers improve bioavailability and enhance the drug effect while reducing the required dose and frequency of administration. The end result is a decrease in the toxicity and side effects for both injection and inhalation formulations [80,81,82,83].

Conjugation or encapsulation of antibiotics in polymer and lipid systems for oral administration allows control of the site of release, protects drugs from the aggressive environment of the gastrointestinal tract, and improves intestinal permeability and absorption. These improvements are especially important for polymyxins because they provide control over the release site, rate, and profile, thereby increasing the bioavailability of these drugs [84,85].

Topical administration of polymyxins is limited by their low activity due to their susceptibility to environmental conditions (hydrolysis, oxidation) and the wound environment (pH, proteolysis), in addition to their minimal residence time. Structured gels and polymer nanofibers and membranes can improve the chemical and physical stability of antibiotics, as well as prolong the drug release. Furthermore, these systems are biodegradable and atraumatic, making them suitable topical delivery systems for the treatment of infected skin injuries (acute and chronic wounds, burns, etc.) [86,87,88,89].

Transdermal delivery is also an attractive route for systemic drug administration due to the circumvention of first-pass hepatic metabolism, increased bioavailability, and reduced toxicity, as well as patient convenience. In addition, in the event of side effects, the drug admission into the systemic circulation can be quickly stopped simply by patch removal. The conventional forms for transdermal delivery are patches and films; in addition, the microneedle technology is an innovative approach that overcomes the skin barrier for hydrophilic drugs [90,91,92,93,94].

### 4.1. Polymeric Nano- and Microparticle Carriers

Several different polymeric nano and microparticles have been successfully used for the delivery of polymyxin. In general, these polymeric materials have unique physicochemical and biological properties, including suitable and controllable size and charge, large specific surface area, functionalizable structures, resistance to chemical and enzymatic destruction, ability to penetrate biological barriers, and affinity for the membranes of bacterial cells. Most importantly, the nano and microparticles have the capacity to target drug delivery to sites of infection, thereby reducing drug doses and eliminating many side effects [4,7,95,96,97,98,99,100].

Polymyxins are positively charged at a neutral pH due to the presence of amine functional groups in their structures; therefore, these molecules are able to interact with anionic and polyanionic molecules to form polyelectrolyte complexes. The polymyxin molecules also contain a lipophilic fatty acyl tail and a hydrophilic head group, so they can form micellar structures with anionic polymers [36,101].

Polysaccharides (such as hyaluronan, sodium alginate, starch) and polyaminoacids are the most commonly used materials for polymyxin delivery systems as they are biocompatible, biodegradable, and non-toxic but still provide suitable drug loading efficiency and controlled drug release. Cationic polymers (e.g., chitosan) are sometimes included to coat these particles to stabilize the resulting complexes, as well as to control the surface charge and colistin release [101,102,103,104]. For example, Yasar et al. [101] developed polycomplexes based on anionic starch (molecular weight (MW) > 100,000) and oligochitosan (MW 5000) for the delivery of colistin and tobramycin. Both tobramycin and colistin were loaded during the formation of the starch/chitosan particles (i.e., the chitosan solution was added to a mixture of starch and antibiotic). The most stable anionic polyplexes were produced using a starch/oligochitosan ratio of 10:1. These polycomplexes had a size of 170–380 nm, a surface charge from −17 mV to −30 mV, an encapsulation efficiency (EE) of 97–99%, and loading efficiencies (LE) of 17% (for colistin) and 3% (for tobramycin). The drug release into phosphate-buffered saline (PBS, pH 7.4) reached 20–40% in 16 h. The antimicrobial activity of antibiotic-loaded polycomplexes against *E. coli* and *P. aeruginosa* was similar to that of the corresponding free drugs.

Coppi et al. [105,106,107] designed microparticles (100 nm to 3 μm in size) consisting of alginate (MW 147,000) and chitosan (MW 70,000) for oral administration of polymyxin B. Microparticles that were resistant to gastrointestinal media were prepared from polymyxin–alginate by a spray-drying technique and cross-linking with Ca^2+^ ions, followed by coating with a chitosan solution at a mass ratio polymyxin–chitosan of 1:1. The EE and LE were about 47% and 12%, respectively. The release of polymyxin in 2 h was 20–25% at pH 3.0 and 50–60% at pH 7.4. The microparticles maintained their antibiotic activity against *E. coli* (minimal inhibitory concentration (MIC) 0.625–5.00 μg/mL) but showed reduced antibiotic cytotoxicity against Vero cell cultures. A Caco-2 cell monolayer model study further showed that microparticles were endocytosed by the cells by 6 h of incubation. An ex vivo study showed that the fabricated particles could be taken up by M cells.

Liu et al. [108] developed nanoparticles of colistin (8 nm in size) by complexation of colistin with polyglutamic acid and subsequent stabilization with 1,2-dimyristoyl-sn-glycero-3-phosphoethanolamine-*N*-[methoxy (polyethylene glycol)-2000] ammonium salt (MW 2700). In vitro and in vivo studies showed that the colistin nanoparticles had equivalent antimicrobial activity against *K. pneumoniae* and *A. baumannii* to that of free colistin, but the particles showed improved safety and lower hepatotoxicity than the free drug when administered to mice over a seven-day period (the maximum tolerated dose increased 1.3-fold compared to free colistin).

Zashikhina et al. [109] synthesized amphiphilic charged copolymers of amino acids (hydrophilic lysine and glutamic acid, and hydrophobic phenylalanine) to form interpolyelectrolyte complexes with cationic and anionic peptides, including polymyxin B. The resulting particles were stabilized by coating with cationic or anionic polysaccharides (chitosan, heparin, alginate). The coated particles had a size of 200–2000 nm and a surface charge ranging from −60 mV to 40 mV. The EE was 75–90%, and the LE was 105–630 μg/mg. Drug release in PBS (pH 7.4) over 24 h was 26–60% and 15–30% for noncoated and coated particles, respectively. The coatings also decreased the cytotoxicity and increased the stability of the resulting peptide delivery systems in biological media.

Synthetic polyanionic macromolecules have also been used to design polymyxin delivery systems. For example, Costa et al. [110] fabricated poly-butyl-cyanoacrylate-based nanoparticles of polymyxin B (217 nm in size, with a surface charge of −18 mV), and found them effective in the treatment of leishmaniasis and associated Gram-negative bacterial infections. The release profile of polymyxin from the nanoparticles (into PBS, pH 7.4) was 50% of the loaded drug in 6 h, which was 6–7 times slower than observed with free polymyxin. An in vitro evaluation of the leishmanicidal activity indicated that the number of uninfected macrophages reached approximately 85%, with practically no highly infected macrophages. The MIC against *P. aeruginosa* for both encapsulated polymyxin and free polymyxin was 2 μg/mL.

Low molecular weight anionic molecules can also be used to produce polyelectrolyte particles of colistin. Abouelmagd et al. [111] studied the formation of pH-sensitive delivery systems for colistin via electrostatic interaction using the anionic polyphenolic compound tannic acid. The resulting particles had a negative surface charge and ranged in size from 1.5 to 5 μm, depending on the ratio of tannic acid/antibiotic. The EE was 50–60%, and the LE was 20–40%. The colistin release was 90–100% in 2 h at pH 4.5 and from 30% to 40% in 2 h at pH 7.4. The fabricated complexes had a MIC approximately two-fold higher than that of free colistin (0.6 and 8 μg/mL against *E. coli* and *S. aureus*, respectively) at pH 7.4, and the complexes showed MIC levels comparable to those of free drugs (0.3 and 4 μg/mL against *E. coli* and *S. aureus*, respectively) at pH 4.5. Overall, the developed systems appeared suitable for the targeted delivery of drugs to inflammatory tissues.

Among inorganic materials, mesoporous silica can be used to produce colistin nanoparticles. The resulting nanoparticles have high porosity for highly effective drug loading. The surface functionalization of mesoporous silica nanoparticles with molecular or polymer moieties provides them with great potency for controlling drug delivery [112,113]. The effectiveness of silica nanoparticles for polymyxin B delivery has been studied by Gounani et al. [114,115], who loaded mesoporous silica nanoparticles and carboxyl-modified mesoporous silica nanoparticles with polymyxin B as well as polymyxin B and vancomycin for the treatment of polymicrobial infections. The fabricated systems had a size of 70–130 nm, and they released 50% of both antibiotics in 24 h, with an 80% release in 80 h (into PBS, pH 7.4). The nanoparticles were effective against both Gram-negative and Gram-positive bacteria, and their efficacy superseded the activity of the free drugs, changing the effect from bacteriostatic to bactericidal at 1 × MIC concentration.

In summary, the delivery of polymyxins can be improved by encapsulating them in carriers based on anionic biopolymers or complexes of anionic and cationic polymers. In this case, the modification of antibiotic release is regulated by the MW of the polymers, the component ratios, particle sizes, and preparation methods.

### 4.2. Lipid-Based Nanostructures (Liposomes and Niosomes)

Liposome-mediated drug delivery is used to overcome the barriers to cellular and tissue uptake of drugs in both parenteral and oral administration. Liposomes are highly effective against microbial biofilms as they easily penetrate and accumulate throughout the entire thickness of the biofilm [116,117]. The potential use of liposomes for encapsulating polycationic polymyxins is hindered by their phospholipid membrane permeability. The research into polymyxin effects on artificial membrane models has shown various effects, ranging from increasing surface roughness to pore formation and leakage of contents [118]. The preparation of stable liposomal formulations with controlled sustained release often involves surface modification of the liposomes with anionic lipids. In addition, the integrity of bilayer membranes is controlled by the amount of loaded polymyxin [118,119,120,121,122].

Li et al. [119] loaded colistin into liposomes modified with sodium cholesteryl sulfate for intravenous administration. The phospholipid membrane permeability to colistin was reduced by modifying the liposomes with sodium cholesteryl sulfate. This improves the colistin loading by increasing the colistin–bilayer electrostatic interaction. The resulting liposomes had a size of 100–200 nm, ζ-potential of −60 to −66 mV, and EE of 50%. Colistin release (into 10 mM CaCl_2_) was 15–44% in 24 h. The pharmacokinetics results showed an approximately four-fold increase in the plasma AUC (0–8 h) for fabricated liposomes when compared with a free colistin solution.

Wang et al. [120] encapsulated colistin and ciprofloxacin in anionic liposomes. Incorporation of anionic lipids (1,2-dimyristoyl-sn-glycero-3-phosphoglycerol and hydrogenated soybean phosphatidylcholine) into the liposomes increased the EE of colistin compared to its EE in neutral liposomes (67.0% versus 18.4%). At least 70% of the loaded colistin was associated with the lipid bilayer on the outer surface of the liposomes, while about 30% of the loaded colistin was most likely located within the lipid membrane and oriented by its hydrophilic part toward the inner aqueous phase. The liposomal formulation had sizes of approximately 100 nm and EEs of 67.0% (for colistin) and 85.2% (for ciprofloxacin). In vitro release of ciprofloxacin and colistin into bovine serum for 2 h was greater than 70% and 50%, respectively. Liposome-encapsulated drugs showed enhanced in vitro antimicrobial activities against multidrug-resistant *P. aeruginosa* when compared to the free drugs.

Wallace et al. [121] obtained liposomes of colistin or colistin methanesulfonate to use in pulmonary inhalation. The prepared carriers had a size of 160–190 nm and EE of 50%. Colistin liposomes had a positive charge (+15 mV), and colistin methanesulfonate liposomes were anionic (−20 mV), indicating a direct association of the amphiphilic polypeptide with the liposome membrane. Investigation of the colloidal stability of the colistin-loaded liposomes showed that the particle size and ζ-potential were stable over the seven days. The particle size and ζ-potential of the colistin methanesulfonate-loaded liposomes were stable only for 48 h, and then a substantial growth in particle size occurred, together with a charge reversal from negative to positive. This instability and the subsequent sedimentation of colistin methanesulfonate-loaded liposomes were explained by the conversion of colistin methanesulfonate to colistin. The release of both colistin and colistimethate into PBS (pH 7.4) was rapid (50% of the total content in 10 min) and remained constant throughout the 72 h experiment.

Yu et al. [122] prepared colistin and ciprofloxacin co-loaded liposomes using mannitol, sucrose, and leucine as formulations for a dry powder inhaler. The EE values of colistin and ciprofloxacin were 47% and 45%, respectively. A cytotoxicity study demonstrated that the liposomal formulations were not cytotoxic at the drug concentrations of 5 μg/mL colistin and 20 μg/mL ciprofloxacin. The liposomal formulation showed superior antibacterial activity against *P. aeruginosa* when compared to each antibiotic alone.

Menina et al. [118] developed liposomes for oral delivery of colistin for the treatment of intracellular infections by *Salmonella enterica*. The stability of the liposomes was improved by the inclusion of cholesterol (30 mol%), which increased the integrity of vesicles by promoting alignment of the phospholipid alkyl chains and by enabling a condensed packing of the lipid bilayer. In addition, gastrointestinal-resistant liposomes were prepared using saturated long-carbon-chain phospholipids (1,2-dipalmitoyl phosphatidylcholine, 1,2-dipalmitoyl-sn-glycero-3-phosphoethanolamine-*N*-(glutaryl), and 1,2-distearoyl-sn-glycero-3-phosphocholine). The size of the liposome formulations ranged from 200 to 700 nm, and the negative surface charge ranged from −10 to −30 mV; the EE was 55–62%, while the LE was about 50%. Notably, the EE was inversely proportional to the colistin concentration. This phenomenon can be explained by the ability of colistin to disturb liposomal membranes. Thus, an increase in colistin concentration may lead to disruption of the liposomal structure and a reduced capacity for drug incorporation. Colistin release into biorelevant media (Fasted State Simulated Intestinal Fluid and Fed State Simulated Intestinal Fluid) was 20–50% in 5 h. Cell experiments (HEp-2 and Caco-2 cells infected with *S. enterica*) showed a significant reduction of intracellular bacteria after liposome treatment when compared with free colistin.

Niosomes are also used to improve antibiotic delivery. A niosome is a non-ionic vesicle that is formed mostly from a non-ionic surfactant, with cholesterol incorporated as an excipient. Niosomes can incorporate both hydrophilic drugs (in an aqueous layer) and lipophilic drugs (in a vesicular lipid membrane). The niosomes are used as delivery systems for poorly absorbable drugs and they increase drug absorption and bioavailability by penetration of the GIT barrier through transcytosis through M cells [123]. For example, Chauhan and Bhatt [124] developed polymyxin B niosomes using sorbitan monostearate (Span 60) and cholesterol to improve intestinal permeability. The fabricated niosomes had a size of 150–750 nm, their surface charge varied from −14 to −31 mV, and the EE was 45–80%. The niosomes protected polymyxin from the GIT environment and increased its absorption in the intestine through M cells. Analysis of rat creatinine concentration as a test of renal toxicity indicated that the creatinine concentration was within normal limits and nephrotoxicity was comparable to that of polymyxin B sulfate injection (500,000 units). In this case, the use of niosomes as a polymyxin delivery system improved its bioavailability by oral administration.

### 4.3. Conjugates

Conjugation of active pharmaceutical substances with various compounds can change the physicochemical properties of drug molecules or generate prodrugs with a sustained and controlled release. In addition, this type of modification of antimicrobial agents can reduce microbial resistance [125,126,127,128,129].

A polymyxin-cinnamaldehyde conjugate was obtained by the creation of an imine bond between the primary amino groups of polymyxin and the carbonyl group of cinnamaldehyde. This increased the drug lipophilicity and its subsequent incorporation into lipophilic nanoparticles, such as self-emulsifying drug delivery systems for oral administration. At the same time, the molecule polarity (log P) was increased 69-fold. The release of polymyxin ranged from 45% to 81% within 16 h [130].

Dextrin–colistin conjugates were synthesized using a carbodiimide coupling reaction with a colistin content of 7.6% *w/w*. The fabricated conjugates exhibited lower antimicrobial activity against *E. coli* than was observed for unmodified colistin (7–12 log-fold reduction in MIC); however, dextrin conjugation reduced colistin in vitro toxicity toward human kidney proximal tubule cells (HK-2, the IC50 concentration improved by four- to five-fold) [131]. Dextrin–colistin conjugates reduced the toxicity of colistin and improved targeting to sites of bacterial infection due to effective binding with bacterial lipopolysaccharide.

In biological studies, both colistin and the dextrin–colistin conjugate effectively inhibited lipopolysaccharide-induced hemolysis and tumor necrosis factor-alpha secretion, but only the dextrin–colistin conjugate showed no additive toxicity at high concentrations [132]. A study of the antibacterial activity of dextrin–colistin conjugate against *A. baumannii* showed a prolonged antimicrobial effect for up to 48 h, while free colistin was active for only 4 h. An in vivo study showed that colistin was effectively released from the conjugate by endogenous alpha-amylase within a wound environment (up to 86.3% at 48 h) [133]. Colistin–dextrin conjugates exhibited comparable antimicrobial activity to colistimethate sodium against Gram-negative pathogens (*A. baumannii*, *E. coli*, *K. pneumoniae*, and *P. aeruginosa*), but in vitro toxicity toward kidney cells was significantly reduced (the IC50 increased by four times). In vivo dose-escalation studies in rats demonstrated improved prolonged pharmacokinetics of the conjugates (t_1/2_ 130–1300 min vs. 53 min) and decreased toxicity when compared to colistin sulfate [134].

A poly(ethylene glycol) methyl ether acrylate (PEGA-480) bioconjugate of colistin has been synthesized via copper-mediated, photoinduced, living radical polymerization using the initiator linker 2-(2-bromo-2-methylpropanoyloxy) acetic acid (degrees of polymerization of 5, 10, and 20). The initial colistin release (into PBS, pH 7.4) was 30–40% in 5 h, and the total release occurred by 48 h. In vitro experiments showed that the conjugate’s antimicrobial activity against *A. baumannii* was similar to or better than that of the clinically relevant colistin prodrug colistimethate sodium [135].

Gold nanoparticles were functionalized with colistin using polyethylene glycol as a linker for later use in binding *A. baumannii*. The resulting particles had an average size of 20 nm and a surface charge of about −6 mV. In vitro studies showed that particle complexation with *A. baumannii* occurred rapidly and reached half-maximum saturation in 7 min [136].

### 4.4. Structured Gels (Hydrogels and Microgels)

Various structured gels based on natural and synthetic polymers are used to develop sustained release antimicrobial dosage forms for the treatment of wounds and burns [137,138,139,140]. Hydrogels and microgels are dispersions of loosely crosslinked polymeric colloids that can be used as delivery systems for polypeptides due to their swelling behavior. They can protect peptides from chemical and enzymatic degradation and increase physical stability by reducing aggregation or conformational changes [141].

For example, phenylboronic acid-functionalized polycarbonate hydrogels loaded with polymyxin B demonstrated controlled in vitro drug release kinetics (75–100% in 50 h) and in vitro antimicrobial activity against *P. aeruginosa* over 48 h (MIC = 2 μM, determined by the disc diffusion method), as well as in vivo antimicrobial efficacy against *P. aeruginosa* burn wound infections [142].

A dextran-poly(ethylene glycol) hydrogel covalently conjugated with polymyxin B and vancomycin has been designed as a wound dressing. The releases (into PBS, pH 7.4) of polymyxin B and vancomycin were 9.2% and 19.7%, respectively, on the first day, and the values remained almost unchanged for the first two weeks. The hydrogel exhibits potent antibacterial activities against both Gram-negative (*E. coli*) and Gram-positive (*S. aureus*) bacteria. In addition, the hydrogels facilitated the penetration of the drug into the bacterial cell membrane and killed bacteria without a cytotoxic effect on the NIH 3T3 mouse fibroblast cell line [143].

Chitosan hydrogels with zinc phthalocyanine-colistin conjugates demonstrated improved efficiency against *P. aeruginosa* when compared to free colistin in an in vitro study [144]. Microfluidics-based self-assembled alginate microgels crosslinked with Ca^2+^ were loaded with polymyxin B to give an EE above 80%. The size of the microgel particles was 100–200 nm and the ζ-potential ranged from −10 to −30 mV. The polymyxin release from the obtained microgels was 20–30% in 10 mM Tris buffer; however, an increase in the ionic strength to 150 mM NaCl resulted in 100% polymyxin release within 1 h [145].

### 4.5. Polymeric Nanofibers and Membranes

Electrospinning technology is used to produce non-woven fiber mats. The electrospun fibers have a large specific surface, excellent mechanical performance, porous structure, and unique nanometer-scale architecture. Therefore, these materials have great potential for use as antibacterial wound dressings [146,147,148,149,150]. For example, Zhang et al. [151] prepared poly(l-lactide) electrospun mats for loading polymyxin B and dexamethasone. The drug release (into tris-HCl buffer, pH 7.4) from the electrospun mats was 23% (for dexamethasone) and 30% (for polymyxin B) for 35 days. An in vitro study confirmed the antibacterial activity of the developed systems against Gram-positive and Gram-negative bacteria, as the mats completely inhibited the growth of *E. coli* over a 110 h period, and the *S. aureus* inhibition efficiencies were 98.8% and 49.5% after 16 and 28 h, respectively. An in vivo study of infected full-thickness burns and infected wounds demonstrated healing on day 14.

Huang et al. [152] prepared a polymyxin B-immobilized nanofiber sponge for endotoxin adsorption by electrospinning and freeze-drying technology. The endotoxin removal rate of a polymyxin-free nanofiber sponge was about 17%, while the polymyxin-immobilized nanofiber sponge adsorbed 99% of the endotoxin. The endotoxin removal rate in human plasma was 90%, and the adsorption reached equilibrium within 60 min, thereby confirming the great potential of polymyxin-immobilized-nanofiber sponges for clinical blood purification.

Polymer membranes can also be used as colistin delivery systems for treating wounds and burns. An elastomer nanocomposite membrane loaded with polymyxin B in halloysite nanotubes with ciprofloxacin has been developed for wound dressing. The membrane released 50% of the polymyxin in 20 h, and 80–100% in 120 h (into PBS, pH 7.4). All the nanocomposites exhibited antimicrobial activity against both Gram-negative (*P. aeruginosa*) and Gram-positive (*S. aureus*) bacteria in more than seven days due to sustained drug release. This drug system is highly water-absorbing and shows low cytotoxicity, good biodegradability, and appreciable elasticity [153].

### 4.6. Microneedles

Microneedle technology is a transdermal, but minimally invasive, delivery system for active pharmaceutical substances, including antimicrobial agents, peptides, and proteins. Microneedles penetrate the stratum corneum and dissolve into the intercellular fluid of the skin to ensure systemic drug delivery [154,155,156,157].

Dillon et al. [156] developed a microneedle system composed of polyvinylpyrrolidone and trehalose and encapsulated polymyxin B for transdermal delivery. The disc diffusion method showed the effectiveness of fabricated microneedles against *Salmonella typhimurium*. Ex vivo skin diffusion studies showed the system successfully delivered antibiotics through porcine skin during the first 4 h of application at a faster initial rate than a drug-loaded control disc.

## 5. Future Aspects

The toxicity of polymyxins observed in the clinic is due to their antimicrobial mechanism, which involves interaction with and damage to bilayer membranes. The same mechanism, however, can cause similar and significant damage to the membranes of cells that make up human organs, like the liver and kidney, when polymyxins are administered at high doses. Therefore, dose reduction is a prime concern in the development of safe and effective polymyxin formulations for targeted and controlled drug release. The development of polymyxin dosage forms should be based on the combined achievements of chemistry, nanotechnology, and nanomedicine. Solving polymyxin delivery and toxicity problems will require chemical conjugation of the drug molecules with biocompatible and biodegradable materials, as well as the development of new compositions based on nanoparticles, liposomes, microneedles, and composite nanomaterials.

Currently, polymeric and lipid-based particles with different parameters may be used to improve both oral and parenteral administration of polymyxin via targeted and control release, while also reducing dose and toxicity. These approaches require a search for safe, biocompatible, and biodegradable materials. Undoubtedly, natural polymers and their derivatives will prove to have great medical potential in this respect. Further research is, therefore, needed to develop systems based on natural polymers (e.g., polyelectrolyte complexes, self-assembled particles, electrospray particles, etc.).

Conjugation of polymyxins is a promising method for improving their intestinal permeability and absorption, as well as for preventing microbial resistance to this vital antimicrobial agent. In general, the chemical modification of polymyxin molecules is insufficiently studied; and information is extremely sparse regarding the effect of modification of polymyxin molecules on drug stability, antimicrobial activity, and the ability to overcome microbial resistance. At the moment, the threat of global resistance to polymyxins is a major problem in the treatment of Gram-negative multiresistant infections, since polymyxins are the last-line drugs. Chemical modification of existing drugs may, therefore, prove to be the solution to the drug resistance problem and may represent the future of antimicrobial drug chemistry.
